# Exploring Zinc C295 as a Dual HIV-1 Integrase Inhibitor: From Strand Transfer to 3′-Processing Suppression

**DOI:** 10.3390/ph18010030

**Published:** 2024-12-29

**Authors:** Sharif Karim Sayyed, Marzuqa Quraishi, D. S. Prabakaran, Balaji Chandrasekaran, Thiyagarajan Ramesh, Satish Kumar Rajasekharan, Chaitany Jayprakash Raorane, Tareeka Sonawane, Vinothkannan Ravichandran

**Affiliations:** 1 Amity Institute of Biotechnology, Amity University Maharashtra, Mumbai 410206, Maharashtra, India; sayyedsharif22@gmail.com (S.K.S.); marzuqa20@gmail.com (M.Q.); tareekasonawane@gmail.com (T.S.); 2 TAQGEN, Molecular Virology Lab of Hootone Remedies, Mumbai 400050, Maharashtra, India; 3 Department of Biotechnology, School of Bioengineering, SRM Institute of Science and Technology, Kattankulathur 603203, Tamil Nadu, India; prabakad3@srmist.edu.in (D.S.P.); satishkr2@srmist.edu.in (S.K.R.); 4 Department of Biotechnology, Ayya Nadar Janaki Ammal College (Autonomous), Srivilliputhur Main Road, Sivakasi 626124, Tamil Nadu, India; 5 Department of Pharmaceutical Sciences, College of Pharmacy, Texas A&M University, College Station, TX 77845, USA; bchandrasekaran@tamu.edu; 6 Department of Basic Medical Sciences, College of Medicine, Prince Sattam Bin Abdulaziz University, Al-Kharj 11942, Saudi Arabia; r.thiyagarajan@psau.edu.sa; 7 School of Chemical Engineering, Yeungman University, Gyeongsan 38541, Republic of Korea; 8 Center for Drug Discovery and Development (CD3), Amity Institute of Biotechnology, Amity University Maharashtra, Mumbai 410206, Maharashtra, India

**Keywords:** drug resistance, dual inhibition, HIV-1 integrase inhibitors, in vitro assays, molecular docking simulation, antiretroviral drug development

## Abstract

**Background**: The global AIDS pandemic highlights the urgent need for novel antiretroviral therapies (ART). In our previous work, Zinc C295 was identified as a potent HIV-1 integrase strand transfer (ST) inhibitor. This study explores its potential to also inhibit 3′-processing (3′P), thereby establishing its dual-targeting capability. **Methods**: The inhibitory activity of Zinc C295 against 3′P was evaluated using a modified in vitro assay adapted from our earlier ST inhibition studies. Molecular docking and molecular dynamics simulations were employed to analyse Zinc C295’s interactions with the 3′P allosteric site of HIV-1 integrase. **Results**: Zinc C295 demonstrated significant inhibition of HIV-1 integrase 3′P activity in in vitro assays (IC50 = 4.709 ± 0.97 µM). Computational analyses revealed key interactions of Zinc C295 within the enzyme’s allosteric site, providing insights into its dual inhibitory mechanism. **Conclusions**: Zinc C295’s dual inhibition of HIV-1 integrase ST and 3′P establishes it as a promising candidate for next-generation ART. Its dual-action mechanism may offer potential advantages in enhancing treatment efficacy and addressing drug resistance. Further studies are warranted to evaluate its therapeutic potential in clinical settings.

## 1. Introduction

Human Immunodeficiency Virus (HIV), the causative agent of Acquired Immunodeficiency Syndrome (AIDS), remains a significant global health challenge, affecting an estimated 38 million people worldwide [1]. This persistent epidemic underscores the critical need for novel antiretroviral therapies [2]. While current antiretroviral therapy regimens have significantly improved patient outcomes, challenges such as drug resistance and long-term side effects necessitate the continued exploration of new therapeutic strategies [3,4,5,6,7].

HIV-1 integrase is a key enzyme in the viral replication cycle and a prime target for developing antiretroviral therapies (ART). Integrase (IN) catalyzes the integration of viral complementary DNA (vcDNA) into the host genome, a crucial step for viral replication and the establishment of chronic infection. This integration process involves two key enzymatic activities: 3′-processing (3′P), in which IN removes two nucleotides from the 3′ ends of the vcDNA, and strand transfer, where IN inserts the processed vcDNA into the host chromosome. Current IN inhibitors primarily target the ST step, leaving 3′P as a relatively underexplored target for therapeutic intervention [8,9,10].

Natural compounds have emerged as promising candidates in HIV drug discovery due to their structural diversity and potential to target multiple stages of the viral life cycle [11,12,13,14,15]. In our previous work [16], the authors identified Zinc C295 as a potent inhibitor of HIV-1 IN ST. Zinc C295 exhibited superior efficacy to other natural molecules, targeting the Mg^2+^-binding DDE motif within the IN active site, similar to clinically approved integrase strand transfer inhibitors (INSTIs) like Raltegravir and Dolutegravir. Furthermore, its unique binding characteristics and robust pharmacophore profile were confirmed through molecular simulations and pharmacokinetic analyses, along with significant in vitro inhibitory activity.

This study focuses specifically on investigating the inhibitory activity of Zinc C295 against HIV-1 IN 3′P, building upon our previous findings of its ST inhibition. This dual-targeting approach offers the potential for enhanced therapeutic efficacy and a reduced likelihood of drug resistance development. The authors hypothesize that Zinc C295’s ability to inhibit both 3′P and ST will provide a more comprehensive and effective approach to HIV treatment. This study aims to characterize Zinc C295 as a multifunctional HIV-1 inhibitor, with a particular emphasis on its 3′P inhibitory activity, to support the development of next-generation ART.

## 2. Results and Discussion

### 2.1. Dual Inhibition Analysis (3′-Processing Assay)

The HIV-1 integrase 3′P inhibition assay is a crucial tool for developing ART that targets the integration of vcDNA into the host genome [17,18]. This study evaluated the inhibitory potency of Zinc C295 against HIV-1 IN 3′P activity across a range of concentrations (0.5 µM to 20 µM). The PI % and the half-maximal inhibitory concentration values (in both µM and µg mL^−1^) are summarized in Table 1.

Zinc C295 demonstrated a clear dose-dependent inhibition of HIV-1 IN 3′P activity. Even at the lowest concentration (0.5 µM), a modest but measurable inhibition (8.93%) was observed. The inhibitory effect increased significantly with increasing concentrations, reaching 73.71% at 20 µM. The calculated IC50 value of 4.709 µM (2.085 µg mL^−1^) indicates a potent inhibitory effect. This suggests that Zinc C295 has a strong affinity for the IN enzyme and effectively disrupts the 3′P processing step of viral replication.

Zinc C295, previously identified as an HIV-1 strand transfer inhibitor, is explored in this study for its inhibition of the 3iP integrase allosteric site, targeting the LEDGF/p75 binding site. Its mechanism involves inhibiting the 3′P activity of integrase, offering a novel approach to modulating integrase function. This observed potency aligns with previous studies highlighting the effectiveness of non-natural compounds, such as bifunctional quinolonyl-diketo acid derivatives and L-731,988, as potent INSTIs. These compounds exhibit high antiviral activity while maintaining moderate toxicity [19]. Another promising category, allosteric integrase inhibitors (ALLINIs), targets the LEDGF/p75 binding site to induce integrase hyper-multimerization, disrupting viral particle morphogenesis and preventing the formation of mature, infectious virions, thereby enhancing therapeutic effects [20,21].

The ongoing emergence of drug-resistant HIV strains underscores the need for the continued development of novel ART. The favourable inhibitory profile of Zinc C295, coupled with the ADME analysis supporting a favourable pharmacokinetic profile, makes it a promising candidate for further development as a next-generation ART. Its potential use as a standalone or adjunctive agent in combination therapies could optimize antiretroviral efficacy, potentially reduce drug resistance, and minimize adverse effects. Further in vitro and in vivo studies are needed to fully explore its clinical potential, especially in combination with other ART agents targeting different stages of the HIV life cycle.

### 2.2. Anti-HIV Strand Transfer (ST) Activity

Strand transfer is a critical step in the HIV-1 integration process, where the vcDNA is incorporated into the host genome by the HIV-1 IN enzyme [16,18]. Inhibiting this step is crucial for disrupting the HIV replication cycle, potentially preventing the virus from establishing a persistent infection [19]. Targeting both the 3′P and ST steps offers the possibility of synergistic antiviral effects, reducing the likelihood of drug resistance and improving treatment outcomes. Evaluating compounds that inhibit these integration steps is essential for developing therapies aimed at disrupting this key aspect of the HIV lifecycle.

**Figure 1:**
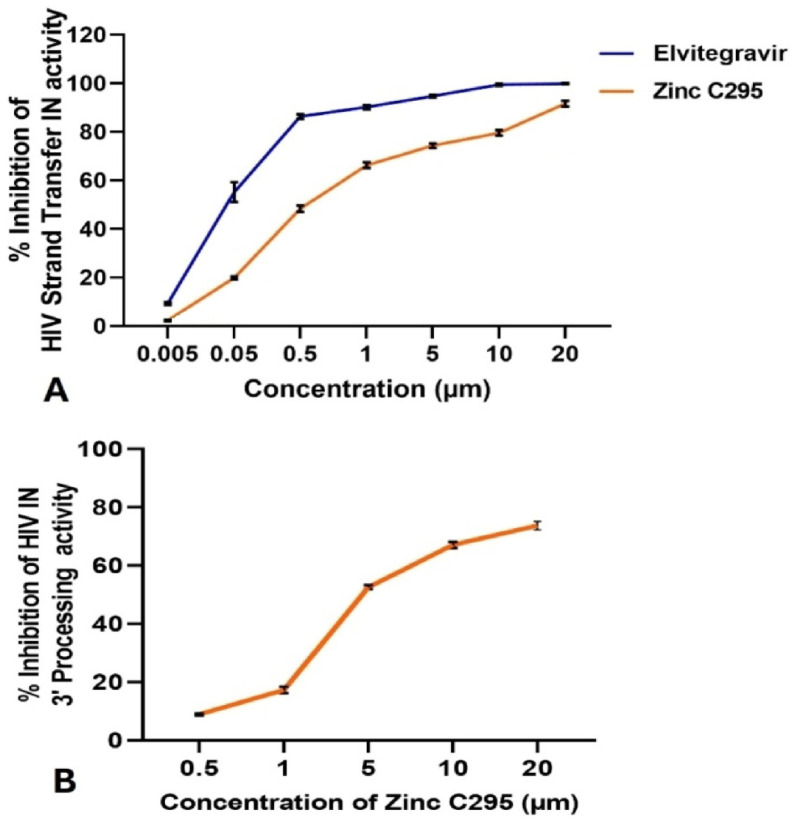
Percent inhibition of HIV-1 (**A**) strand transfer (ST) activity and (**B**) integrase 3′ processing (3′P) activity.

Figure 1A shows the PI % of ST activity by different compounds. Elvitegravir (ELV) exhibited the highest inhibition, approaching nearly 100% at higher concentrations. Zinc C295 also demonstrated strong, dose-dependent inhibition, with 79.59% inhibition at 10 µM. This level of inhibition compares favourably with other reported inhibitors, suggesting its potential as an effective antiviral agent.

As shown in Figure 1B, Zinc C295 demonstrated 67.11% inhibition in the 3′P assay at a concentration of 10 µM. These findings underscore its dual inhibitory activity against both 3′P and strand transfer (ST) processes. This inhibitory profile suggests that Zinc C295 holds significant potential as a candidate for further development in HIV therapy.

The IC50 values for Zinc C295 in the strand transfer and 3′P assays were 0.546 µM (0.241 µg mL^−1^) and 4.709 µM (2.085 µg mL^−1^) respectively, demonstrating potent inhibitory activity. The dose-dependent inhibition observed in both assays further supports its potential as a therapeutic agent.

Our findings are consistent with previous research emphasizing the importance of targeting HIV-1 integrase as an effective therapeutic strategy. Zinc C295, which inhibits 3′P activity, demonstrated significant inhibition in our assays, similar to findings reported by Panwar et al. Their study identified the human Lens Epithelium-Derived Growth Factor (LEDGF/p75) as a key cofactor in viral replication and explored potential inhibitors that disrupt the IN-LEDGF/p75 interaction [22]. Their research highlighted compounds like ZINC22077550 and ZINC32124441 as potent HIV inhibitors with strong binding affinity and favourable pharmacological profiles. Furthermore, studies on LEDGINs have provided deeper insights into the inhibition mechanisms of HIV-1 integrase. For example, molecular modelling of LEDGINs such as BI-1001 and CX14442 revealed that these compounds engage the HIV-1 integrase catalytic core domain via hydrophobic interactions and hydrogen bonding, with CX14442 showing enhanced affinity due to its larger tert-butyl group interacting with the IN CCD dimer interface. These findings underscore the significance of understanding binding modes and allosteric inhibition mechanisms, which can contribute to the design of more effective anti-HIV agents [23].

Additionally, research into IN-LEDGF allosteric inhibitors, such as Mut101, highlights their dual activity in inhibiting both IN-LEDGF interaction and IN strand transfer activity, impacting both the integration and post-integration stages of the viral replication cycle [24]. Our study also emphasizes the growing importance of ALLINIs, which target non-catalytic sites on HIV-1 integrase, inducing aberrant enzyme multimerization [25,26,27]. This approach offers a potential advantage in combating HIV resistance by targeting a mechanism distinct from current therapies, thereby reducing the likelihood of cross-resistance. Pyridine-based ALLINIs, like STP0404, which bind to the LEDGF/p75 pocket, have shown efficacy in disrupting viral replication [28]. The efficacy of these compounds suggests that targeting non-catalytic sites, a potential mechanism of action for Zinc C295 could offer a promising approach to addressing HIV resistance. This strategy may help prevent viral integration even in the presence of mutations that confer resistance to other classes of inhibitors. However, further research and additional assays are required to confirm whether Zinc C295 operates through this mechanism and to assess its potential to overcome resistance in clinical settings.

Further studies support the potential of allosteric HIV-1 IN inhibition, which is central to our investigation of Zinc C295. Screening compounds from the ZINC15 database has revealed binding sites at the IN-dimer interface, offering insights into potential allosteric mechanisms. For example, HDS1 targets the DNA binding step of HIV-1 IN integration [29], a process that is closely linked to the 3′P step the authors are investigating. Both steps are critical for the incorporation of vcDNA into the host genome, and inhibiting either can disrupt the integration process. This highlights the importance of targeting key protein-DNA interactions, a concept relevant to our study of Zinc C295, which similarly interacts with integrase and potentially disrupts its DNA binding activity.

Additionally, the identification of 18 natural compounds targeting the IN-LEDGF/p75 interface, with potent compounds exhibiting IC50 values of 0.32 and 0.26 μM, further supports the potential of natural compounds as effective anti-HIV agents. For instance, NPD170, which shows an EC50 of 1.81 μM, inhibits HIV integration by interfering with the integrase-LEDGF/p75 interaction [30]. The efficacy of these natural compounds strengthens our findings regarding the inhibitory activity of Zinc C295, suggesting that targeting critical protein-protein or protein-DNA interfaces is a viable strategy and that natural compounds, such as Zinc C295, can serve as potent inhibitors of HIV-1 integrase.

Figure 1 presents data from the 3′-processing inhibition assay at 10 µM, demonstrating that Zinc C295 significantly inhibits HIV-1 IN activity, exhibiting 67.11% inhibition at this concentration. Sodium azide, a known chelator, was used as a positive control in this study. Although no direct control drug was used in the 3′P assay, the inhibition observed with Zinc C295 at 10 µM suggests strong potential for its development as an effective integrase inhibitor. The substantial reduction in integrase activity emphasizes its role in inhibiting the 3′P step, which is crucial for HIV-1 integration.

### 2.3. Molecular Docking Analysis

**Figure 2:**
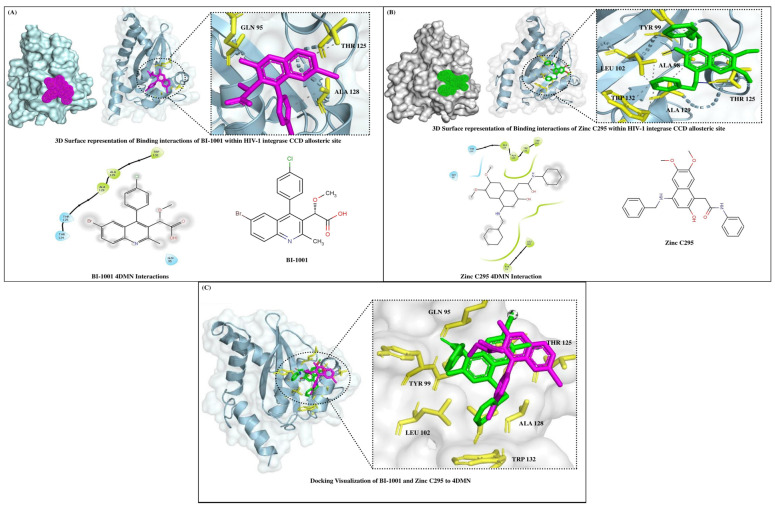
Visualization of molecular docking interactions between BI-1001 and Zinc C295 with the HIV-1 IN catalytic core domain (CCD) allosteric site (PDB ID: 4DMN). The figure presents 3D representations of the docking interactions generated using PyMOL and 2D representations created with Maestro, along with the ligand structures drawn in Marvin: (**A**) BI-1001 (magenta), (**B**) Zinc C295 (green), and (**C**) the combined interactions of BI-1001 and Zinc C295 with HIV-1 IN. In the 2D interaction diagram, the residues involved in hydrogen bonds are indicated in blue, including Gln95, Thr124, and Thr125, while the residues engaged in hydrophobic interactions are shown in green, such as Trp132, Ala128, and Ala129. Key binding interactions are highlighted to illustrate the molecular interactions involved in the binding efficacy of both ligands.

The 2D and 3D interactions between HIV-1 IN and natural molecules were analyzed using molecular docking. Figure 2 illustrates the binding interactions of BI-1001 and Zinc C295 within the CCD allosteric site. The three-dimensional surface representation shows the spatial fit of BI-1001 within the binding pocket, highlighting the ligand-enzyme interaction regions and the overall complementarity between the ligand and the enzyme’s active site. The interacting residues are shown in yellow and magenta, which correspond to BI-1001, indicating its stable and favourable binding conformation (Figure 2A).

The reference ligand BI-1001 has been extensively studied as a benchmark for evaluating potential integrase inhibitors. BI-1001 binds to the HIV-1 IN CCD allosteric site, where it forms key interactions that contribute to its potent inhibitory action. Notably, BI-1001 establishes hydrogen bonds with Glutamine 95 (Gln 95) and Threonine 124 (Thr124), with additional hydrogen bonding at Threonine 125 (Thr125). These polar interactions are further complemented by hydrophobic contacts with residues such as Alanine 128 (Ala128), Alanine 129 (Ala129), and Tryptophan 132 (Trp132), which stabilize the ligand’s binding within the allosteric site (Figure 2A). This strong interaction network plays a critical role in the efficacy of BI-1001 as an integrase inhibitor, contributing to its ability to disrupt HIV replication by blocking integrase function.

Zinc C295, the compound under investigation in this study, exhibits a similar, highly effective binding profile within the same allosteric site of HIV-1 integrase, engaging several key residues that are comparable to those targeted by BI-1001. Like BI-1001, Zinc C295 forms a hydrogen bond with Glutamine 95 (Gln95), anchoring the ligand firmly within the allosteric site. Threonine 125 (Thr125) also participates in hydrogen bonding with Zinc C295, contributing to the specificity and stability of its binding. While BI-1001 interacts with both Threonine 124 and Threonine 125, Zinc C295 interacts primarily with Threonine 125, achieving a stable binding configuration with slightly fewer polar contacts compared to BI-1001, but still maintaining high specificity for the binding site (Figure 2B).

In terms of hydrophobic interactions, both BI-1001 and Zinc C295 engage with critical residues such as Alanine 128 (Ala128), Alanine 129 (Ala129), and Tryptophan 132 (Trp132), stabilizing their respective binding conformations within the allosteric site. However, Zinc C295 extends its hydrophobic interactions to additional residues, including Tryptophan 131 (Trp131), Tyrosine 99 (Tyr99), and Leucine 102 (Leu102), which further stabilize the ligand and contribute to its optimized binding profile within the integrase allosteric site. These additional interactions likely enhance the stability and inhibitory potency of Zinc C295.

The binding profile of Zinc C295 is highly comparable to that of BI-1001, supporting its potential as an effective HIV-1 integrase inhibitor (see Figure 2C). Both ligands utilize a similar mechanism of action, with key hydrogen bonds and hydrophobic interactions stabilizing their binding within the integrase allosteric site. Zinc C295’s slightly extended hydrophobic interaction network may offer an advantage in terms of binding affinity and inhibitory potency. These findings suggest that Zinc C295 could be a promising candidate for further development as an allosteric modulator of HIV-1 integrase, with the potential to inhibit both the 3′P and ST steps of integrase function.

The binding profile of Zinc C295 closely mirrors that of BI-1001, reinforcing its potential as a potent HIV-1 integrase inhibitor. Zinc C295’s interactions with both the CCD and residues critical for integrase dimerization align with the mechanisms of established allosteric integrase inhibitors (ALLINIs), such as GSK1264, which disrupt integrase function by targeting the CCD dimer interface and inducing aberrant multimerization [31]. Similarly, thiophene-carboxylic acid derivatives, known to target the CCD-CCD dimer interface at the LEDGF/p75 binding pocket, emphasize the importance of this site in inhibitor design [32].

Our findings are consistent with earlier studies demonstrating that ALLINIs not only induce inappropriate integrase multimerization but also inhibit both the catalytic domain and the IN-LEDGF/p75 interaction [33,34]. Zinc C295’s dual-targeting mechanism suggests its potential to disrupt both 3′ processing (3′P, allosteric site) and ST reactions of HIV-1 IN, key steps in viral DNA integration.

The structural interactions of Zinc C295, including its engagement with critical hydrophobic and polar residues, support its classification as a promising novel inhibitor. This compound offers the potential to develop innovative therapeutic strategies targeting HIV-1 integrase. Future studies are essential to fully elucidate its mechanism of action and therapeutic efficacy in inhibiting HIV replication.

### 2.4. Molecular Dynamics (MD) Simulation

**Figure 3:**
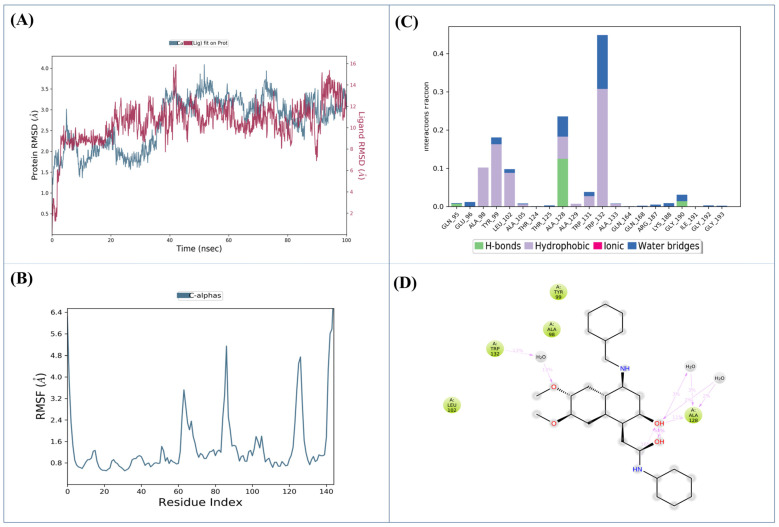
MD simulation analysis of the Zinc C295-4DMN complex over 100 ns. (**A**) **RMSD plot** showing the stability and fluctuations of the 4DMN protein and Zinc C295, with protein RMSD ranging from 1.5 to 4 Å, indicating overall structural stability. (**B**) **RMSF plot** identifying flexible regions, particularly around residue indices 60, 70, and 130, suggesting potential ligand interaction sites. (**C**) **Bar graph** illustrating the fraction of time key residues, including Ala133, Ala98, Tyr99, and Leu102, interacted with Zinc C295, highlighting hydrophobic contacts. (**D**) **Ligand–protein contact map** detailing interactions, including hydrogen bonds and water-mediated contacts, with residues such as Ala128 and Trp132, contributing to the stability of the Zinc C295-4DMN complex.

MD simulations were performed to evaluate the stability and interaction profile of Zinc C295 in complex with the HIV-1 IN CCD. The RMSD plot (Figure 3A) demonstrates the stability of the Protein-ligand complex over 100 ns of simulation. The protein’s RMSD values fluctuated between 1.5 and 4.0 Å, indicating overall structural stability. The system stabilized after ~20 ns, with only minor deviations observed, suggesting that the Zinc C295-IN complex maintained a robust binding conformation throughout the simulation.

The RMSF plot (Figure 3B) highlights the flexibility of individual residues in the IN protein. Most residues exhibited low RMSF values, below 2.0 Å, consistent with a stable protein structure. However, peaks were observed at residue indices around 60 (3.2 Å), 70 (5.6 Å), and 130 (6.4 Å), corresponding to regions of increased flexibility. These regions, likely loops or disordered segments, may facilitate ligand accommodation and optimize Protein-ligand interactions. The terminal ends of the protein showed higher fluctuations, as expected, while the core regions between indices 0–50 and 90–120 remained relatively rigid and stable.

Figure 3C illustrates the interaction profile between Zinc C295 and the IN protein during the simulation. Alanine 133 (Ala133) demonstrated the highest interaction frequency, maintaining stable contact with Zinc C295 for approximately 40% of the simulation time. Other significant interactions were observed with residues such as Alanine 98 (Ala98), Tyrosine 99 (Tyr99), Leucine 102 (Leu102), Alanine 128 (Ala128), and Tryptophan 132 (Trp132), which formed hydrophobic interactions critical for stabilizing the ligand within the allosteric site. Hydrogen bonding interactions were noted with residues such as Glutamine 95 (Gln95), Glutamate 96 (Glu96), Alanine 105 (Ala105), and Threonine 124 (Thr124), although these were less frequent, indicating dynamic interactions.

These findings underscore the stability and efficacy of Zinc C295 in binding the HIV-1 IN CCD allosteric site. The combination of hydrophobic and hydrogen-bonding interactions, particularly involving residues Ala133, Ala128, and Trp132, contributes significantly to the ligand’s inhibitory potential. Flexibility in loop regions near the binding pocket further facilitates ligand accommodation and conformational adjustments, enhancing the binding profile. This robust interaction network supports the previously observed inhibitory activity of Zinc C295 in both ST and 3′P assays, positioning it as a promising candidate for further development in anti-HIV therapy.

Ligand–protein contact data (Figure 3D) reveals several critical interactions between Zinc C295 and the HIV-1 IN CCD. Alanine 128 (Ala128) forms hydrogen bonds and water-mediated bridges with the ligand, while Tryptophan 132 (Trp132) predominantly engages in water-mediated interactions. Additionally, Tyrosine 99 (Tyr99), Leucine 102 (Leu102), and Alanine 98 (Ala98) exhibit both direct and occasional water-mediated contact with Zinc C295. These hydrophobic interactions play a pivotal role in stabilizing the Protein-ligand complex by anchoring the ligand firmly within the binding pocket, thereby reducing the likelihood of dissociation and enhancing binding affinity. The network of consistent and strong interactions is crucial for the specificity and stability of the complex, underscoring their importance in drug-receptor dynamics and the development of effective HIV-1 integrase inhibitors.

The previous literature emphasizes the role of hydrophobic interactions in the binding affinity and efficacy of INSTIs. For instance, Proline 145 (Pro145) has been identified as a key hydrophobic residue in the active site of HIV-1 integrase, with studies demonstrating that hydrophobic interactions are essential for inhibitor binding and overcoming drug resistance [35,36]. Similarly, Chang et al. [37] highlighted the significance of hydrophobic contacts in the binding of GRL-02031 to HIV-1 protease, showcasing the broader importance of these interactions in stabilizing enzyme-inhibitor complexes. While focused on protease, the findings align with the role of hydrophobic interactions observed in the Zinc C295-HIV-1 IN interaction.

Hydrogen-bonding patterns also contribute significantly to inhibitor efficacy. Barecca et al. [38] explored the impact of hydrogen bonds within the integrase active site on binding stability and resistance. Their study on the 5CITEP inhibitor revealed the critical role of hydrogen bonds in maintaining a stable interaction profile, consistent with the patterns observed for Zinc C295 [39]. Additionally, the catalytic loop of HIV-1 integrase, encompassing residues 138–149, has been identified as a dynamic region amenable to inhibitor targeting. Specific residues, such as Glutamine 62 (Gln62), and the role of water molecules in bridging ligands to the enzyme’s active site, have been shown to contribute to binding dynamics, further supporting the potential of Zinc C295 to effectively interact with flexible and conserved regions of IN [40].

Collectively, the ligand-protein interaction data and the corroborative findings from the literature emphasize the robust binding profile of Zinc C295. Its ability to engage in both hydrophobic and hydrogen-bonding interactions, coupled with water-mediated bridges, positions it as a promising candidate for further exploration as a novel HIV-1 integrase inhibitor.

### 2.5. ADME Analysis

Zinc C295 adhered to Lipinski’s rules, indicating favourable oral bioavailability and potential as a promising drug candidate (see Table 2). Its molecular mass of 442.51 Da falls within the acceptable range for successful oral drugs. Compounds with molecular weights below 500 Da generally exhibit better permeability and absorption, enhancing bioavailability due to easier cellular membrane passage and reduced steric hindrance [38,41].

The ESOL Log S value classifies Zinc C295 as moderately soluble, supporting effective absorption in the body. Optimal solubility is essential for drug absorption, distribution, and therapeutic efficacy, ensuring the compound reaches target sites at therapeutic concentrations [42,43]. Zinc C295 has 9 H-bond donors and 4 H-bond acceptors, which suggests a potential for forming stable interactions with target proteins. However, excessive H-bonding can sometimes hinder permeability. Its LogP value of 3.23 indicates moderate hydrophobicity, which balances solubility and permeability, supporting its drug-like properties [44,45].

The ligand exhibits high GI absorption, a critical factor for oral administration. However, it is not predicted to permeate the BBB, limiting its applicability to central nervous system (CNS) disorders. Achieving BBB permeability is recognized as a significant challenge in drug discovery [45,46]. The bioavailability score of 0.55 indicates moderate potential for bioavailability [47]. Furthermore, adherence to Lipinski’s rules with zero violations reinforces its favourable pharmacokinetic profile [47,48]. Literature underscores the importance of these parameters in identifying drug candidates with optimal absorption, distribution, metabolism, and excretion (ADME) characteristics [49,50,51].

**Figure 4:**
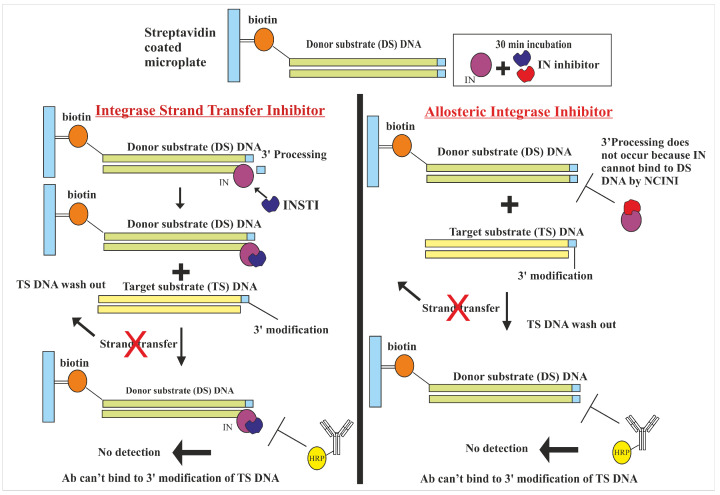
Mechanism of HIV Integrase Inhibition. This schematic illustrates two types of integrase inhibitors: strand transfer inhibitors (INSTIs) (**left**) and allosteric integrase inhibitors (**right**). INSTIs inhibit the IN enzyme by preventing the joining of the target substrate DNA to the donor substrate DNA following the 3′ processing (3′P) step. In contrast, allosteric inhibitors act by blocking the 3′ processing step itself, thereby preventing IN from binding to DS DNA and interfering with the integration process.

Based on our comprehensive in vitro and in silico studies, the authors propose the mechanisms of action for our lead compound, Zinc C295, as illustrated in the figures provided. Figure 4 compares two classes of HIV-1 IN inhibitors: INSTIs and allosteric integrase inhibitors. Zinc C295, acting as an INSTI, prevents the IN enzyme from catalyzing the joining of the target substrate (TS) DNA with the donor substrate (DS) DNA after the 3′P step. In contrast, allosteric inhibitors target the 3′P step itself, preventing IN from binding to the DS DNA, thereby blocking the critical action in the integration process.

### 2.6. Summary of Dual Inhibition Potential of Zinc C295

HIV infection involves a series of steps, beginning with viral entry into host cells and the conversion of viral RNA into vcDNA. A key enzyme in this process is IN, which facilitates the integration of vcDNA into the host genome. IN performs two crucial reactions: 3′ processing (3′P), where it removes nucleotides from the 3′ ends of the vcDNA, and strand transfer (ST), where it integrates the vcDNA into the host genome. Targeting these steps offers a promising approach to inhibit HIV replication and improve therapeutic outcomes.

**Figure 5:**
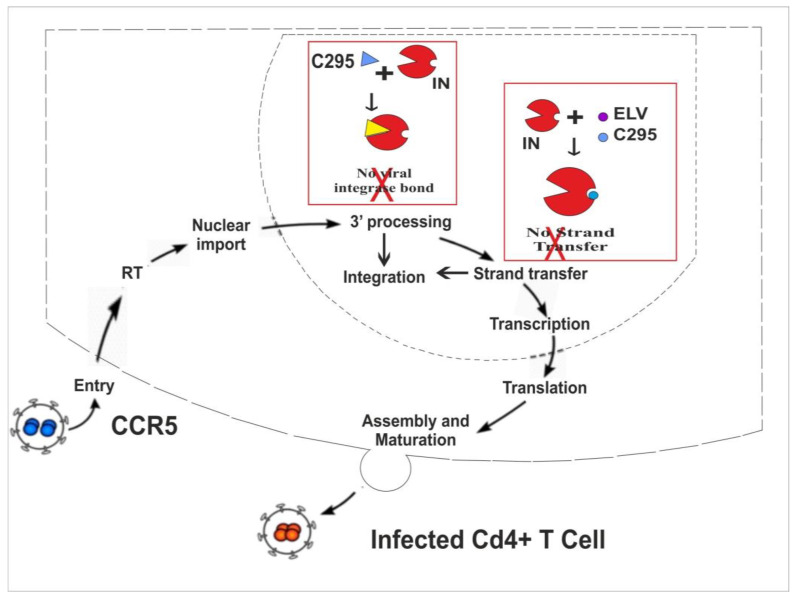
Schematic representation of HIV infection with inhibitory effects demonstrated by the proposed lead compounds against stages of IN (strand transfer and 3′ processing). Abbreviations: C295 is Zinc C295, ELV is Elvitegravir.

In this study, the authors investigated the inhibitory potential of Zinc C295, a novel compound, on the HIV-1 integrase enzyme. Zinc C295 effectively inhibited both 3′P and ST reactions of IN, demonstrating significant promise as a dual-target inhibitor (refer to Figure 5).

These findings suggest that Zinc C295 holds significant potential in anti-HIV integrase research. Future studies should further explore its mechanism of action and assess its therapeutic potential in clinical settings. Future studies should further explore its mechanism of action and assess its therapeutic potential in clinical settings. Further in vitro studies using the TZM-bl cell line are recommended, wherein the cells would be treated with Zinc C295 and subsequently analyzed for integrated and un-integrated proviral DNA using a quantitative PCR protocol.

## 3. Materials and Methods

### 3.1. HIV-1 IN 3′-Processing Inhibition Assay

3′-Processing (3′P) inhibition assay was performed using the HIV-1 Integrase Assay Kit (XpressBio, Frederick, MD, USA) with minor modifications. Initially, 96-well plates were coated with 100 µL of donor substrate DNA corresponding to the HIV-1 LTR region and incubated overnight at 4 °C. After incubation, the wells were washed three times with PBS to remove unbound DNA, followed by blocking with 200 µL of PBS containing 5% BSA for 1 h at room temperature. This step ensured the elimination of nonspecific binding.

HIV-1 integrase (20 nM) was pre-incubated with serial dilutions of the inhibitor (ranging from 0.05 to 20 µM) prepared in Dimethyl sulfoxide (or DMSO with final DMSO concentration ≤ 5%) for 30 min at 37 °C. Sodium azide served as a positive control, while a no-inhibitor condition and a DMSO-only control were included as the negative and solvent controls, respectively. The integrase-inhibitor mixtures (100 µL) were then added to the DNA-coated wells and incubated for 1 h at 37 °C. Following incubation, the wells were washed with PBS to remove unbound integrase and inhibitors.

For detection, 100 µL of HRP-conjugated anti-integrase antibody was added to each well and incubated for 1 h at 37 °C. After washing five times with PBS, 100 µL of tetramethylbenzidine (TMB) substrate solution was added and allowed to react for 10 min at room temperature. The reaction was stopped with 100 µL of TMB stop solution, and absorbance was measured at 450 nm using a microplate reader. IC50 values were determined using GraphPad Prism.

$$Percent\;inhibition\;\left(PI\%\right)=\frac{Mean\;absorbance\;of\;Control-Mean\;absorbance\;of\;test\;articles}{Mean\;absorbance\;of\;Control}\times 100$$

### 3.2. Computational Analysis for Dual Inhibition

#### 3.2.1. Model Preparation

The crystal structure of the HIV-1 integrase catalytic core domain complexed with the allosteric inhibitor BI-1001 (PDB ID: 4DMN) was used for computational analysis. This structure was selected because it is a high-resolution structure that clearly defines the 3′P allosteric binding site and includes a bound inhibitor, making it ideal for understanding inhibitor interactions and facilitating structure-based drug design. The protein was prepared using Biovia Discovery Studio Visualizer (v16.1.0.15350), during which crystallographic water molecules and ligands were removed. Hydrogen atoms were added to simulate physiological conditions, and Gasteiger charges were assigned using AutoDock Tools (v1.5.6).

#### 3.2.2. Ligand Preparation

The chemical structure of Zinc C295 was retrieved from the ZINC15 database. Ligand preparation involved adding hydrogen atoms, assigning charges, and optimizing atom types using Biovia Discovery Studio Visualizer (v16.1.0.15350) to ensure compatibility with AutoDock.

#### 3.2.3. Molecular Docking

Molecular docking was performed using AutoDock v4.2 to explore Zinc C295’s binding interactions with the HIV-1 IN 3′P site. A grid box was defined around the allosteric site of the catalytic core domain (CCD), centred at x = −41.951205, y = 6.627740, z = 18.384534 with dimensions of 20 × 20 × 20 Å, encompassing key residues Thr124, Thr125, and Ala128. The Lamarckian Genetic Algorithm was employed as the search algorithm, and 10 docking runs were conducted with an exhaustiveness setting of 10 to ensure comprehensive sampling.

Binding affinities, expressed in terms of binding energy (kcal mol^−1^)**,** were calculated for the lowest energy pose. The interactions between Zinc C295 and the target residues were analyzed using PyMOL and ProteinsPlus. Key interactions included hydrogen bonds, hydrophobic interactions, and π-π stacking, with further characterization of bond lengths and hydrophobic contacts supporting the stability and specificity of Zinc C295 binding at the 3′P site.

### 3.3. Molecular Dynamics (MD) Simulations

Molecular dynamics (MD) simulations were employed to study the stability and dynamics of Zinc C295 in complex with the HIV-1 integrase 3′P site. The optimal docking pose of Zinc C295, selected based on its interaction profile, was used as the input for MD simulations using the Schrödinger Suite 2019-4. Protein–ligand complexes were prepared in Schrödinger Maestro by assigning bond orders, adding hydrogen atoms, and optimizing protonation states at pH 7 under the OPLS3e force field. The systems were solvated in orthorhombic water boxes using the TIP3P water model, maintaining a minimum distance of 10 Å between the complex and the box boundary. Neutralization was achieved using counter-ions (Na^+^ or Cl^−^), and physiological ionic strength was set to 0.15 M NaCl.

Simulation parameters were set up using Schrödinger Suite 2019-4. The equilibration phase included energy minimization using a steepest descent algorithm with convergence criteria of 1 kcal/mol/Å. This was followed by stabilization under the NVT ensemble at 300 K for 1 ns using a Nose-Hoover thermostat and the NPT ensemble at 1 atm for 1 ns using a Martyna–Tobias–Klein barostat. Production MD simulations were performed for 100 ns using the Desmond MD engine with a 2 fs time step, saving snapshots every 100 ps. Temperature was regulated using a Nose-Hoover chain thermostat with a relaxation time of 1 ps, and pressure was maintained using a Martyna–Tobias–Klein barostat with a relaxation time of 2 ps.

Post-simulation analyses were conducted using Schrödinger and included root-mean-square deviation (RMSD) to assess the stability of the protein backbone and ligand, using the initial structure as the reference. Root-mean-square fluctuation (RMSF) was calculated for protein Cα atoms to identify flexible regions within the protein. Protein–ligand contact analyses identified and quantified key stabilizing interactions, including hydrogen bonds, hydrophobic contacts, and salt bridges, using distance and angle cutoffs. The percentage of simulation time these interactions were maintained was also calculated to evaluate their stability throughout the simulation.

### 3.4. ADME (Absorption, Distribution, Metabolism, and Excretion) Profile

The ADME properties of Zinc C295 were assessed in silico to predict its drug-likeness and pharmacokinetic profile. Key properties, including molecular mass, hydrogen bond donors and acceptors, LogP, and molar refractivity, were calculated. Water solubility was predicted using the ESOL model, while gastrointestinal (GI) absorption was evaluated using bioavailability models. The potential for blood–brain barrier (BBB) penetration was assessed as part of its pharmacokinetic profile. Compliance with Lipinski’s rule of five and bioavailability scores were determined to predict its oral bioavailability.

### 3.5. Statistical Analysis

Data from three independent experiments were analyzed using GraphPad Prism (v10.2.3). Statistical significance was assessed using unpaired *t*-tests for comparisons between two groups and one-way ANOVA with a post hoc Tukey’s test for multiple comparisons. A *p*-value less than 0.05 was considered statistically significant.

## 4. Conclusions

The study establishes Zinc C295 as a promising inhibitor of HIV-1 integrase, a crucial enzyme facilitating viral replication. The molecule demonstrated robust inhibitory activity and strong binding interactions, with efficacy comparable to or exceeding that of current reference drugs. Notably, its natural origin and multi-target potential present a novel avenue for addressing key challenges in HIV therapeutics, including drug resistance and the limitations of existing treatment regimens. These findings position Zinc C295 as a candidate with significant therapeutic value, offering potential improvements in clinical outcomes and the overall management of HIV/AIDS.

The integration of Zinc C295 into combination antiretroviral therapies could provide a strategic advantage by targeting a critical stage of the viral lifecycle, thereby enhancing the robustness and efficacy of treatment protocols. This dual approach may also mitigate the emergence of resistant viral strains, a persistent challenge in current HIV management strategies.

Future research should prioritize elucidating the precise molecular interactions of Zinc C295 with HIV-1 integrase through advanced structural and biochemical analyses. Additionally, optimization through structure-activity relationship (SAR) studies and the development of synthetic analogues will be vital to enhance its pharmacological profile. Rigorous preclinical evaluation, encompassing pharmacokinetics, pharmacodynamics, and toxicity assessments, is essential before progressing to clinical trials. These steps will determine its feasibility for integration into existing treatment frameworks and its potential as a stand-alone therapy.

The successful translation of Zinc C295 from bench to bedside could represent a significant leap forward in the fight against HIV/AIDS. By addressing critical gaps in current therapeutic approaches, this research lays the groundwork for innovative solutions that could redefine the standards of care and contribute to global efforts to control and ultimately eradicate HIV.

## Figures and Tables

**Table 1:** HIV-1 IN 3′P inhibition at varying concentrations along with IC50 values in µM and µg mL^−1^.

Concentration (µM)	Zinc C295 (% Inhibition)	*p*-Value
0.5	8.93 ± 0.29	0.0001
1	17.32 ± 1.15	
5	52.56 ± 0.69	
10	67.11 ± 1.04	
20	73.71 ± 1.44	
IC50 (µM)	4.709 ± 0.97	
IC50 (µg mL^−1^)	2.085 ± 0.97	

Data represent the mean inhibition ± standard deviation.

**Table 2:** Molecular properties and drug-likeness prediction of ligands according to Lipinski’s rules using the SwissADME tool.

Parameters	Zinc C295
Molecular Mass	442.51
Hydrogen bond donor	9
Hydrogen bond acceptor	4
LogP	3.23
Molar Refractivity	131.55
ESOL Log S	−5.64
ESOL Class	Moderately soluble
GI absorption	High
BBB Permeant	No
Lipinski violations	0
Bioavailability Score	0.55

## Data Availability

The original contributions presented in this study are included within the article. Further inquiries can be directed to the corresponding authors.

## References

[1] UNAIDS Global HIV & AIDS Statistics—Fact Sheet. https://www.unaids.org/en/resources/fact-sheet.

[2] Khalifa Z., Patel A.B. (2024). Tri-Substituted 1,3,5-Triazine-Based Analogs as Effective HIV-1 Non-Nucleoside Reverse Transcriptase Inhibitors (NNRTIs): A Systematic Review. Drug Dev. Res..

[3] Hasan K., Ferdianti F., Paryati S. (2023). Anti-HIV Transcriptase Herbs: A Review. ACTA Med. Health Sci..

[4] Abela I.A., Scherrer A.U., Böni J., Yerly S., Klimkait T., Perreau M., Hirsch H.H., Furrer H., Calmy A., Schmid P. (2020). Emergence of Drug Resistance in the Swiss HIV Cohort Study Under Potent Antiretroviral Therapy Is Observed in Socially Disadvantaged Patients. Clin. Infect. Dis..

[5] Yu D.H., Wagner S., Schütz M., Jeon Y., Seo M., Kim J., Brückner N., Kicuntod J., Tillmanns J., Wangen C. (2024). An Antiherpesviral Host-Directed Strategy Based on CDK7 Covalently Binding Drugs: Target-Selective, Picomolar-Dose, Cross-Virus Reactivity. Pharmaceutics.

[6] Geng X., Ding X., Zhu Y., Chong H., He Y. (2024). Characterization of Novel HIV Fusion-Inhibitory Lipopeptides with the M-T Hook Structure. Microbes Infect..

[7] Mbhele N., Chimukangara B., Gordon M. (2021). HIV-1 Integrase Strand Transfer Inhibitors: A Review of Current Drugs, Recent Advances and Drug Resistance. Int. J. Antimicrob. Agents.

[8] Garnett G.P. (2020). The Intergenerational Impact of a Slow Pandemic: HIV and Children. New Dir. Child. Adolesc. Dev..

[9] Omer S., Mohamed O., Mohamed H., Ali A., Abdeldaiem E., Ali M., Ali S., Mustafa M., Hassan S., Hassan M. (2020). Knowledge, Attitude, and Testing of Human Immunodeficiency Virus Infection among 15- to 49-Year-Old Women in Sudan: An Analysis of the United Nations Children’s Fund-Multiple Indicator Cluster Survey. Dr. Sulaiman Al Habib Med. J..

[10] Madia V.N., Ialongo D., Messore A., Patacchini E., Arpacioglu M., Scipione L., Di Santo R., Roberta C. Discovery of Quinolinonyl Derivatives as Anti-HIV-1 Inhibitors Endowed with an Innovative Mechanism of Action. Proceedings of the XXVIII Edition of the EFMC International Symposium.

[11] Fauk N.K., Mwanri L., Hawke K., Mohammadi L., Ward P.R. (2022). Psychological and Social Impact of HIV on Women Living with HIV and Their Families in Low- and Middle-Income Asian Countries: A Systematic Search and Critical Review. Int. J. Environ. Res. Public Health.

[12] Mendonca C.J., Newton-John T.R.O., Bulsara S.M. (2022). Psychosocial Factors and Quality of Life in HIV. Aust. Psychol..

[13] Siew Z.Y., Asudas E., Khoo C.T., Cho G.H., Voon K., Fang C.M. (2024). Fighting Nature with Nature: Antiviral Compounds That Target Retroviruses. Arch. Microbiol..

[14] Mandal A., Biswas D., Hazra B. (2020). Natural Products from Plants with Prospective Anti-HIV Activity and Relevant Mechanisms of Action. Stud. Nat. Prod. Chem..

[15] Kaur R., Sharma P., Gupta G.K., Ntie-Kang F., Kumar D. (2020). Structure-Activity-Relationship and Mechanistic Insights for Anti-HIV Natural Products. Molecules.

[16] Sayyed S.K., Quraishi M., Jobby R., Rameshkumar N., Sonawane T., Ravichandran V. (2024). Identification of a Novel HIV-1 Integrase Strand Transfer Inhibitor: A Synergistic Approach Combining Pharmacophore Modelling and In Vitro Assays. ChemistrySelect.

[17] Tran B.X., Phan H.T., Latkin C.A., Nguyen H.L.T., Hoang C.L., Ho C.S.H., Ho R.C.M. (2019). Understanding Global HIV Stigma and Discrimination: Are Contextual Factors Sufficiently Studied? (GAPRESEARCH). Int. J. Environ. Res. Public Health.

[18] Sayyed S.K., Quraishi M., Jobby R., Rameshkumar N., Kayalvizhi N., Krishnan M., Sonawane T. (2023). A Computational Overview of Integrase Strand Transfer Inhibitors (INSTIs) Against Emerging and Evolving Drug-Resistant HIV-1 Integrase Mutants. Arch. Microbiol..

[19] Hazuda D.J., Felock P., Witmer M., Wolfe A., Stillmock K., Grobler J.A., Espeseth A., Gabryelski L., Schleif W., Blau C. (2000). Inhibitors of Strand Transfer That Prevent Integration and Inhibit HIV-1 Replication in Cells. Science.

[20] Engelman A.N. (2019). Multifaceted HIV Integrase Functionalities and Therapeutic Strategies for Their Inhibition. J. Biol. Chem..

[21] Krishnan L., Engelman A. (2012). Retroviral Integrase Proteins and HIV-1 DNA Integration. J. Biol. Chem..

[22] Panwar U., Singh S.K. (2021). In Silico Virtual Screening of Potent Inhibitor to Hamper the Interaction Between HIV-1 Integrase and LEDGF/P75 Interaction Using E-Pharmacophore Modeling, Molecular Docking, and Dynamics Simulations. Comput. Biol. Chem..

[23] Xue W., Liu H., Yao X. (2014). Molecular Modeling Study on the Allosteric Inhibition Mechanism of HIV-1 Integrase by LEDGF/P75 Binding Site Inhibitors. PLoS ONE.

[24] Le Rouzic E., Bonnard D., Chasset S., Bruneau J.M., Chevreuil F., Le Strat F., Nguyen J., Beauvoir R., Amadori C., Brias J. (2013). Dual Inhibition of HIV-1 Replication by Integrase-LEDGF Allosteric Inhibitors Is Predominant at the Post-Integration Stage. Retrovirology.

[25] Elliott J.L., Kutluay S.B. (2020). Going Beyond Integration: The Emerging Role of HIV-1 Integrase in Virion Morphogenesis. Viruses.

[26] Sapp N., Burge N., Cox K., Prakash P., Balasubramaniam M., Thapa S., Christensen D., Li M., Linderberger J., Kvaratskhelia M. (2022). HIV-1 Preintegration Complex Preferentially Integrates the Viral DNA into Nucleosomes Containing Trimethylated Histone 3-Lysine 36 Modification and Flanking Linker DNA. J. Virol..

[27] Bowen N.E., Oo A., Kim B. (2022). Mechanistic Interplay between HIV-1 Reverse Transcriptase Enzyme Kinetics and Host SAMHD1 Protein: Viral Myeloid-Cell Tropism and Genomic Mutagenesis. Viruses.

[28] Krupkin M., Jackson L.N., Ha B., Puglisi E.V. (2020). Advances in Understanding the Initiation of HIV-1 Reverse Transcription. Curr. Opin. Struct. Biol..

[29] Gupta K., Allen A., Giraldo C., Eilers G., Sharp R., Hwang Y., Murali H., Cruz K., Janmey P., Bushman F. (2021). Allosteric HIV Integrase Inhibitors Promote Formation of Inactive Branched Polymers via Homomeric Carboxy-Terminal Domain Interactions. Structure.

[30] Dinh L.P., Sun J., Glenn C.D., Patel K., Pigza J.A., Donahue M.G., Yet L., Kessl J.J. (2022). Multi-Substituted Quinolines as HIV-1 Integrase Allosteric Inhibitors. Viruses.

[31] Al-Mawsawi L.Q., Neamati N. (2011). Allosteric Inhibitor Development Targeting HIV-1 Integrase. ChemMedChem.

[32] Maehigashi T., Ahn S., Kim U.I., Lindenberger J., Oo A., Koneru P.C., Mahboubi B., Engelman A.N., Kvaratskhelia M., Kim K. (2021). A Highly Potent and Safe Pyrrolopyridinebased Allosteric HIV-1 Integrase Inhibitor Targeting Host LEDGF/P75-Integrase Interaction Site. PLoS Pathog..

[33] Quashie P.K., Han Y.S., Hassounah S., Mesplède T., Wainberg M.A. (2015). Structural Studies of the HIV-1 Integrase Protein: Compound Screening and Characterization of a DNA-Binding Inhibitor. PLoS ONE.

[34] Hu G., Li X., Zhang X., Li Y., Ma L., Yang L.M., Liu G., Li W., Huang J., Shen X. (2012). Discovery of Inhibitors to Block Interactions of HIV-1 Integrase with Human LEDGF/P75 via Structure-Based Virtual Screening and Bioassays. J. Med. Chem..

[35] Gupta K., Turkki V., Sherrill-Mix S., Hwang Y., Eilers G., Taylor L., McDanal C., Wang P., Temelkoff D., Nolte R.T. (2016). Structural Basis for Inhibitor-Induced Aggregation of HIV Integrase. PLoS Biol..

[36] Patel D., Antwi J., Koneru P.C., Serrao E., Forli S., Kessl J.J., Feng L., Deng N., Levy R.M., Fuchs J.R. (2016). A New Class of Allosteric HIV-1 Integrase Inhibitors Identified by Crystallographic Fragment Screening of the Catalytic Core Domain. J. Biol. Chem..

[37] Chang Y.C.E., Yu X., Zhang Y., Tie Y., Wang Y.F., Yashchuk S., Ghosh A.K., Harrison R.W., Weber I.T. (2012). Potent Antiviral HIV-1 Protease Inhibitor GRL-02031 Adapts to the Structures of Drug Resistant Mutants with Its P1′-Pyrrolidinone Ring. J. Med. Chem..

[38] Barreca M.L., Lee K.W., Chimirri A., Briggs J.M. (2003). Molecular Dynamics Studies of the Wild-Type and Double Mutant HIV-1 Integrase Complexed with the 5CITEP Inhibitor: Mechanism for Inhibition and Drug Resistance. Biophys. J..

[39] Deng N., Hoyte A., Mansour Y.E., Mohamed M.S., Fuchs J.R., Engelman A.N., Kvaratskhelia M., Levy R. (2016). Allosteric HIV-1 Integrase Inhibitors Promote Aberrant Protein Multimerization by Directly Mediating Inter-Subunit Interactions: Structural and Thermodynamic Modeling Studies. Protein Sci..

[40] Fan X., Zhang F.H., Al-Safi R.I., Zeng L.F., Shabaik Y., Debnath B., Sanchez T.W., Odde S., Neamati N., Long Y.Q. (2011). Design of HIV-1 Integrase Inhibitors Targeting the Catalytic Domain as Well as Its Interaction with LEDGF/P75: A Scaffold Hopping Approach Using Salicylate and Catechol Groups. Bioorganic Med. Chem..

[41] Wang Y., Wang X., Xiong Y., Kaushik A.C., Muhammad J., Khan A., Dai H., Wei D.Q. (2020). New Strategy for Identifying Potential Natural HIV-1 Non-Nucleoside Reverse Transcriptase Inhibitors Against Drug-Resistance: An in Silico Study. J. Biomol. Struct. Dyn..

[42] Sotriffer C.A., Ni H., McCammon J.A. (2000). Active Site Binding Modes of HIV-1 Integrase Inhibitors. J. Med. Chem..

[43] Brigo A., Lee K.W., Fogolari F., Mustata G.I., Briggs J.M. (2005). Comparative Molecular Dynamics Simulations of HIV-1 Integrase and the T66I/M154I Mutant: Binding Modes and Drug Resistance to a Diketo Acid Inhibitor. Proteins Struct. Funct. Genet..

[44] Matsson P., Kihlberg J. (2017). How Big Is Too Big for Cell Permeability?. J. Med. Chem..

[45] Deokate S.C., Deokate A.A., Sangale S.B., Gawade T.P. (2023). Drug Solubility: Importance and Enhancement Techniques. Int. J. For. Multidiscip. Res..

[46] Beig A., Miller J.M., Lindley D., Dahan A. (2017). Striking the Optimal Solubility-Permeability Balance in Oral Formulation Development for Lipophilic Drugs: Maximizing Carbamazepine Blood Levels. Mol. Pharm..

[47] Lipinski C.A., Lombardo F., Dominy B.W., Feeney P.J. (2012). Experimental and Computational Approaches to Estimate Solubility and Permeability in Drug Discovery and Development Settings. Adv. Drug Deliv. Rev..

[48] Lipinski C.F., Maltarollo V.G., Oliveira P.R., da Silva A.B.F., Honorio K.M. (2019). Advances and Perspectives in Applying Deep Learning for Drug Design and Discovery. Front. Robot. AI.

[49] Ciura K., Dziomba S. (2020). Application of Separation Methods for In Vitro Prediction of Blood–Brain Barrier Permeability—The State of the Art. J. Pharm. Biomed. Anal..

[50] Crivori P., Cruciani G., Carrupt P.A., Testa B. (2000). Predicting Blood-Brain Barrier Permeation from Three-Dimensional Molecular Structure. J. Med. Chem..

[51] Martin Y.C. (2014). Applications of Pharmacophore Mapping. Reference Module in Chemistry, Molecular Sciences and Chemical Engineering.

